# ​​Longitudinal Erythronychia Secondary to a Wooden Splinter

**DOI:** 10.7759/cureus.33619

**Published:** 2023-01-10

**Authors:** Travis S Dowdle, Blayne Fenner, Dylan Maldonado, Jeremy Purser, Michelle Tarbox

**Affiliations:** 1 Dermatology, Texas Tech University Health Sciences Center School of Medicine, Lubbock, USA; 2 Dermatology, Texas Tech University Health Sciences Center, Lubbock, USA

**Keywords:** dermoscopy, subungal keratosis, dermatopathology, splinter, longitudinal erythronychia

## Abstract

Longitudinal erythronychia (LE) is a term for red streaks in the nail which can be caused by a range of diseases. The specific type of longitudinal erythronychia can correlate with certain associated conditions making it important to properly categorize when discovered. A 71-year-old Hispanic male presented to the clinic with a type 1A LE associated with subungual keratosis that had been asymptomatic for approximately 12 months. The patient denied injury, pain, cold sensitivity, or cosmetic distress. The working diagnosis was squamous cell carcinoma in situ (SCCIS) vs. onychopapilloma or glomus tumor. A 4mm punch biopsy from the distal nail matrix was performed, and dermatopathology revealed that the LE was secondary to a wooden splinter. After a literature review, it was discovered that this is the first confirmed case of LE secondary to a splinter. Future providers should keep splinters as a potential differential diagnosis, especially as they evaluate LE, but ultimately all suspicious type IA lesions should be biopsied to rule out potential insidious pathologies, such as SCCIS and malignant melanoma.

## Introduction

Longitudinal erythronychia (LE) is a term for red streaks in the nail, which can be caused by a range of diseases [1-3]. The lesion(s) typically begins within the nail matrix and therefore, clinically originates at the proximal nail fold [4]. The single or multiple red bands transverse the lunula and continue along the nail bed to reach the distal tip of the nail plate. LE is more common on the fingers than on the toes [5]. The specific type of longitudinal erythronychia can correlate with certain associated conditions making it important to properly categorize when discovered. LE is classified by the number of nails involved and the number of linear bands observed: type 1A is a monodactylous-single band; type 1B are monodactylous-bifid bands; type 2A is a polydactylous-single band; and type 2B are polydactylous-multiple bands [1]. For example, type 1A LE has been most associated with benign subungual neoplasms such as a glomus tumor or onychopapilloma and, more rarely, squamous cell carcinoma in situ (SCCIS) or malignant melanoma [1]. Polydactylous LE is primarily associated with underlying dermatologic or systemic diseases like acantholytic epidermal bullosa, Darier disease, or lichen planus [1,5].

## Case presentation

**Figure 1:**
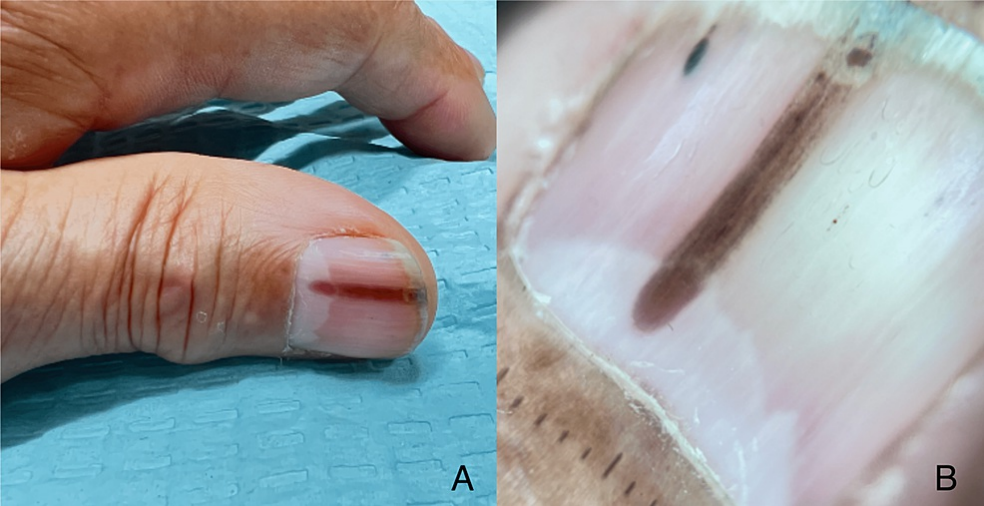
(A) Clinical photo of the longitudinal erythronychia; (B) 10x magnification of the longitudinal erythronychia beginning at the lunula of the nail Magnification of dermoscopy: Dermlite DL4 polarized (Dermlite, San Juan Capistrano, California), 10x

A 71-year-old Hispanic male presented to the Veterans Association (VA) clinic with an asymptomatic type 1A longitudinal erythronychia (LE). It was approximately 2mm in width on his left first digit, beginning at the level of the lunula and extending to the distal tip of the onychodermal band (Figure 1).

**Figure 2:**
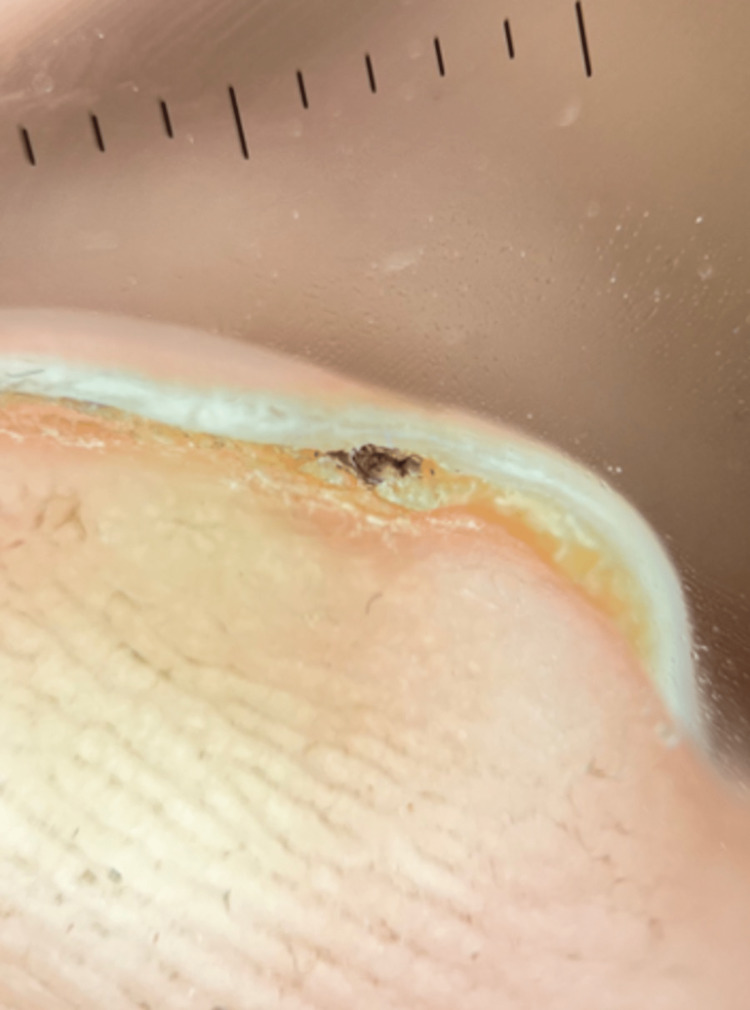
Subungual keratosis at the level of the hyponychium Magnification of dermoscopy: Dermlite DL4 polarized, 10x

He reports the LE had been present for approximately 12 months. The patient denied any previous injury, focal pain, sensitivity to cold, or distress due to cosmetic appearance. During dermoscopy, a subungual keratosis was discovered at the level of the hyponychium (Figure 2).

**Figure 3:**
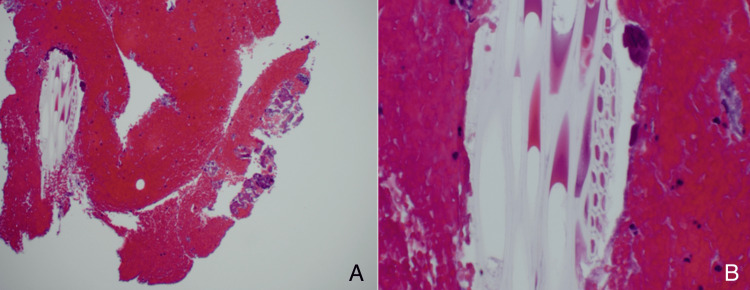
(A) H&E staining of longitudinal section of nail unit, low power view (40x); (B) H&E stain, high power (200x); individual cell walls indicative of plant material (wood) are present

The working diagnosis was squamous cell carcinoma in situ (SCCIS) vs. onychopapilloma or glomus tumor. No breaking of the nail at the free edge, onycholysis, splinter hemorrhages, splitting of the nail, thinning, ridging, or V-shaped distal nicks were observed. Pinpoint tenderness with palpation was absent, negative Love's sign, and disappearance of pain after exsanguination of the digit was absent, and there was a negative Hildreth's sign. No other relevant general medical history of note. A 4mm punch biopsy was obtained from the distal nail matrix down to the periosteum. Dermatopathology revealed fragments of plant material within the dermis beneath the nail bed consistent with a wooden splinter, suggesting that the LE was secondary to a prominent splinter (Figure 3). The adjacent nail matrix was unremarkable, with no dysplasia present. 

## Discussion

The pathogenesis of LE includes a focal loss of function in the distal nail matrix. This can occur secondary to pressure on the matrix (ex: tumor) or from dermatosis-related matrix disease (ex: lichen planus) [1,2]. As a result of matrix function loss, a ventral groove on the underside of the nail plate and a streak of thinned nail within the longitudinal axis are created. This leads to the perception of more nail bed blood vessels due to the thin streak of nail. Also, vessel engorgement occurs in the nail bed that is trapped within the ventral groove [2,3]. The distal edge of the free nail is the oldest, thinnest, and subject to the most trauma, which may account for secondary features observed, such as splitting or other disintegration [1]. 

A PubMed, Scopus, Embase, and Google Scholar search was performed, and this is the first recorded case of LE secondary to a splinter. One of the diagnostic clues could have been the fact that the LE began at the level of the lunula rather than the level of the proximal nail fold; however, due to the patient's asymptomatic nature, a more insidious disease process needed to be ruled out [3,5]. The subungual keratosis as the level of the hyponychium is also highly associated with a glomus tumor as the underlying cause of the LE [1]; however, pinpoint tenderness with palpation was not present, there was a negative Love's sign, and disappearance of pain after exsanguination of the digit was also not present, with a negative Hildreth's sign [3]. The punch biopsy performed in the distal nail matrix reduced the chances of dystrophic nail complications that could occur if the punch was taken more proximally. Dermatopathology revealed fragments of plant material within the dermis beneath the nail bed consistent with a wood splinter (Figure 3).

Future providers should keep splinters as a potential differential diagnosis, especially as they evaluate LE, but ultimately all suspicious type IA lesions should be biopsied to rule out potential insidious pathologies, such as SCCIS and malignant melanoma [5].

## Conclusions

Longitudinal erythronychia can be associated with a range of cutaneous conditions and systemic diseases, making it important to properly categorize and diagnose. Although this is the first documented case of LE secondary to a splinter, in the future, providers should consider splinters as a potential differential diagnosis when working up LE. However, all suspicious type IA lesions should be biopsied in order to rule out squamous cell carcinoma, malignant melanoma, onychopapilloma, glomus tumor, or other insidious pathologies. Dermoscopy and dermatopathology are helpful in distinguishing the different pathologies. 
